# The Genomics of Colorectal Cancer: State of the Art

**DOI:** 10.2174/138920208783884865

**Published:** 2008-03

**Authors:** Andrew D Beggs, Shirley V Hodgson

**Affiliations:** 1 Section of Medical Genetics, St. Georges University of London, London, UK; 2 Department of Colorectal Surgery, Mayday University Hospital, London, UK

**Keywords:** Colorectal, cancer, genomics, epigenomics.

## Abstract

The concept of the adenoma-carcinoma sequence, as first espoused by Morson *et al. *whereby the development of colorectal cancer is dependent on a stepwise progression from adenomatous polyp to carcinoma is well documented.

Initial studies of the genetics of inherited colorectal cancer susceptibility concentrated on the inherited colorectal cancer syndromes, such as Familial Adenomatous Polyposis (FAP) and Lynch Syndrome (also known as HNPCC). These syndromes, whilst easily characterisable, have a well understood sequence of genetic mutations that predispose the sufferer to developing colorectal cancer, initiated for example in FAP by the loss of the second, normal allelle of the tumour supressor APC gene. Later research has identified other inherited variants such as MUTYH (MYH) polyposis and Hyperplastic Polyposis Syndrome.

Recent research has concentrated on the pathways by which colorectal adenomatous polyps not due to one of these known inherited susceptibilities undergo malignant transformation, and determination of the types of polyps most likely to do so. Also, why do individuals in certain families have a predisposition to colorectal cancer.

In this article, we will discuss briefly the current state of knowledge of the genomics of the classical inherited colorectal cancer syndromes. We will also discuss in detail the genetic changes in polyps that undergo malignant transformation as well as current knowledge with regards to the epigenomic changes found in colorectal polyps.

## INTRODUCTION

The model of an adenoma, thought to be initiated by a DNA mutation caused by an environmental agent, promoted by further exposure to the same or a different environmental agent and progression into a carcinoma by a carcinogenic agent was first espoused by Morson *et al. *[1] in 1978. It was also hypothesised in a later paper [2] that certain forms of polyposis had a hereditable component, namely familial adenomatous polyposis, multiple juvenile polyposis and Peutz-Jegher syndrome. 

Although there is strong evidence for the development of carcinoma from a precursor adenomatous polyp there is no definitive evidence for this. It has been estimated that 30-40% of the Western population will develop some adenomas at some stage in their lifetime [3, 4], usually after 40 years of age. Of these patients, only approximately 3% will go on to develop colorectal cancer.

There is a large body of evidence regarding the molecular genetic changes that occur in colorectal carcinogenesis. Initial studies have concentrated on the molecular and genomic changes in easily characterisable inherited colorectal cancer syndromes such as Familial Adenomatous Polyposis (FAP), Lynch Syndrome (also known as Hereditary Non-Polyposis Colorectal Cancer, HNPCC), the more recently characterised MYH polyposis, Peutz-Jegher Syndrome, PTEN Hamartoma Syndrome, Juvenile Polyposis Syndrome and Hyperplastic Polyposis Syndrome.

It has been shown that there are several different series of molecular pathways that benign adenomatous polyps follow in their progression from benign polyp to malignant cancer. These pathways include the Chromosomal Instability pathway (CIN), the Microsatellite Instability Pathway (MSI) and the “Serrated Adenoma” pathway.

## THE INHERITED COLORECTAL CANCER SYNDROMES

### Familial Adenomatous Polyposis (FAP)

FAP is a polyposis syndrome, inherited in an autosomal dominant manner with almost 100% penetrance [5]. It was the first inherited polyposis syndrome to be clinically characterised. It generally presents with total intestinal polyposis within the 2nd – 3rd decade of life. Polyps may be asymptomatic, so that patients may initially present with colorectal tumours.

The incidence of FAP is approximately 1:10,000 [6]. It forms a very small part of the total colorectal carcinoma incidence, estimated at less than one percent. There is no difference between men and women in incidence. 

Patients with FAP present with total colonic polyposis, initially affecting the colon, with the number of polyps increasing over time to between 100-5000. The polyps themselves are adenomatous polyps which are indisguishable from sporadic adenomas [5]. However, the incidence of microadenomas is much greater in patients with FAP [7]. The number of polyps increases the risk of colorectal carcinogenesis greatly, compared to the single adenoma typically found in sporadic cases. The lifetime risk of colorectal carcinogenesis in untreated FAP patients is 100%.

FAP is caused by germline mutations in the *APC* (adenomatous polyposis coli) gene. Usually, affected individuals acquire a defective copy of the *APC* gene from the affected parent, with a somatic mutation of the other copy of the gene causing the initiation of polyposis. However, approximately 30% [5] of cases of FAP present as new germline mutations in the *APC* gene. 

The germline mutations found in the *APC* gene in FAP are very similar to those found in sporadic adenomas and consist of either frameshift or nonsense mutations leading a premature stop codon and truncuation of the *APC* protein product, leading to uncontrolled cellular proliferation. Polyps are initiated by somatic mutation or loss of the normal allele in the susceptible tissue, i.e. colonic epithelium. The mechanism by which mutation of the *APC* gene leads to colorectal carcinogenesis is discussed later in this paper.

Another form of FAP is the attenuated *APC* variant, known as AAPC. This differs from the standard FAP in that although the risk of colorectal carcinogenesis is almost 100%, patients tend to develop polyposis later in life (3rd-5th decade of life) and develop fewer polyps [8].

Mutations in two genes can cause AAPC. The first are mutations in the 3’ and 5’ end of the *APC* gene and in the alternatively spliced exon 9 of the *APC* gene. AAPC may also be caused by mutations in MutYH (human MutY homologue), also known as MYH. Germline mutations in the MYH gene (autosomal recessive inheritance) have been shown to be present in the absence of germline mutations in the *APC* gene in patients with polyposis, and are discussed further below.

### Lynch Syndrome (HNPCC)

Lynch syndrome (also known as Hereditary non-polyposis colorectal cancer syndrome) is a autosomnal dominantly inherited cancer syndrome characterised by early onset of colorectal cancer as well as carcinomas derived from adenomatous tissue such as colorectal endometrial, ovarian, gastric and urinary tract (renal pelvis and ureteric) carcinoma [9, 10]. According to the published literature, it is the commonest form of hereditary colorectal cancer, with an incidence estimated to be between 1-6% of colorectal carcinoma.

The mutations characterised in Lynch Syndrome are in DNA mismatch repair (MMR) enzymes. The MMR genes mutated in the germline in HNPCC have been identified so far as *hMSH2* (located on 2p), *hMLH1* (located on 3p), *hPMS1*, *hPMS2* and *hMSH6*. Over 60% of cases of HNPCC are caused by mutations in *hMSH2* and *hMLH1* [11, 12].

A genetic linkage analysis of Swedish Lynch Syndrome families identified the position of the *MLH1* locus on 3p21 [13, 14]. *hMLH1* encodes the human homologue of the bacterial MutL gene. *PMS1* and *PMS2* are two additional MutL homologues, located on 2q31-33 and 7p22 respectively. Unlike FAP, Lynch syndrome is very rarely caused by a de novo germline mutation in an MMR gene.

*hMSH2* encodes for a human homologue to the bacterial MutS gene and is responsible for mismatch binding [15, 16]. It is part of the complex hMutSα which consists of *MSH2* and *MSH6*, the genes for both are found at 2p21 as shown by FISH studies [17]. It is responsible for repair of base-base mismatches and 1bp insertion/deletion loops. In the hMutSα complex, *MSH6* is responsible for mismatch recognition. 

MLH1 combines with the protein product of *hPMS2* to form the hMutLα complex that acts as an endonuclease involved in the mismatch repair system [18].

The MMR pathway is also thought to be involved in the pathogenesis of sporadic colorectal cancer and is discussed later. 

### MYH Polyposis 

MYH polyposis differs from the classical clinical polyposis phenotype in that although the risk of colorectal carcinogenesis is almost 100%, patients tend to develop polyposis later in life (3rd-5th decade of life) and do not develop as great a number of polyps [8].

This form of AAPC is caused by germline mutations in *MutYH* (human MutY homologue), also known as the *MYH* gene, mutated in the absence of germline mutations in the *APC* gene [19-21]. It is located on 1p32.1.

The *MYH* gene codes for a protein product of a base excision repair enzyme [8]. In the process of normal aerobic metabolism, 7,8-dihydro-8-oxoguanine (8-oxoG) forms that can mis-pair with adenine. This mis-pairing leads to a transversion of G:C to T:A. The *MYH* gene protein product is a DNA glycosylase that excises the abnormal 8-oxoG from the adenine base.

The presence of germline *MYH* gene mutations were first characterised in “Family N” by Al-Tassan *et al. *[19]. They found that three out of seven siblings in the family were affected by an AAPC like phenotype with colorectal carcinoma and polyps in the absence of a germline mutation in *APC* but with somatic mutation of *APC* characterised by G:C to T:A transversions, suggesting a germline mutation leading to a defect of repair of 8-oxoG-related mutations. It was noted that for the phenotype of this mutation to occur; both copies of the defective *MYH* gene had to be inherited, making the inheritance recessive in nature.

Studies of patients with FAP-like and AAPC-like phenotypes with no demonstrable germline mutation in the *APC* gene have shown approximately up to 25% carry bi-allelic mutations of the *MYH* gene [22-24]. In a study by Sieber *et al. *[22] it was found in 152 patients with between 3-100 adenomas that 14 patients had bi-allelic mutations in the *MYH* gene but interestingly no patient who possessed biallelic mutations had severe (>1000) polyposis.

### Peutz-Jegher Syndrome

Peutz-Jegher Syndrome (PJS) is an inherited polyposis syndrome characterised by multiple hamartomatous polyps in the GI tract associated with mucocutaneous pigmentation, especially of the vermillion border of the lips. Its incidence is estimated at approximately 1 in 150,000 [25]. The clinical manifestations occur in the 1^st^-2^nd^ decade of life, and patients present with polyp-related symptoms such as PR bleeding, intussusception, abdominal pain and bowel obstruction [26], although other features such as buccal pigmentation may develop earlier, and may also fade.

A meta-analysis of published data on Peutz-Jegher Syndrome [27] found that the risk ratio for developing any type of cancer was greater than 15, with colonic cancer having a specific risk ratio of 84. 

PJS is due to a germline mutation of the tumour suppressor gene *STK11* [28]. *STK11* encodes a serine-threonine kinase that controls cellular proliferation and also has a role in responding to decreased cellular energy levels [29]. In the regulation of energy levels the protein product of *STK11* acts in a pathway to inhibit AMP-activated protein kinase which then signals downstream to inhibit the mTOR (mammalian target of rapamycin) pathway [30]. This leads to the dysregulation of the mTOR pathway and the initiation of polyposis. 

### PTEN Hamartoma Syndrome (PHTS)

PHTS is a group name given to a group of disorders that are all caused by germline mutations of the tumour suppressor gene *PTEN*(Phosphatase and Tensin homolog) [31]. These disorders consist of Cowden Syndrome, Bannayan-Riley-Ruvalcaba syndrome (BRRS) and Proteus Syndrome.

PTEN hamartoma tumour syndrome is caused by a germline mutation of the *PTEN* tumour suppressor gene [32]. The *PTEN* protein product is a universally expressed phosphatase that has activity against lipid and protein components. In its lipid component, it helps to regulate levels of phosphoinositol triphosphate, providing negative feedback to the AKT pathway. The *PTEN* gene has been shown to have a critical role [33] in the control of cell growth, proliferation and angiogenesis. Further studies have shown that somatic mutations of *PTEN* are present in colorectal malignancies [34]. Gastrointestinal polyps have been shown to be common in Cowden Syndrome, and of varied types [35].

### Juvenille Polyposis Syndrome (JPS)

JPS is defined as a hamartomatous polyposis syndrome affecting both children and, to a lesser extent, adults. To be diagnosed, it requires the presence of greater than 3-5 polyps in the colon or rectum, or juvenile polyps throughout the GI tract or any number of juvenile polyps in an individual who has a family history of JPS [36, 37]. JPS polyps have a characteristic appearance [38] and there are usually multiple polyps in the affected GI tract, presenting usually before 20 years of age [39].

The genetic mutation underlying JPS involves the transforming growth factor-β pathway [40]. Mutations have been described in the *SMAD4*, *BMPR1A *and *ENG* genes. These genes all encode for proteins involved in the TGF-β pathway. As discussed above, the genes *SMAD2* and *SMAD4* are both components of the TGF-β pathway and are involved in colorectal carcinogenesis. In a study by Howe *et al. *[41] there was a 20% prevalence of germline mutations of *BMPR1A* and *SMAD4*. At the present time we do not know the prevalence of germline mutations of *ENG*.

### Hyperplastic Polyposis Syndrome (HPS)

Hyperplastic polyposis syndrome (HPS) is a recently identified, poorly classified entity. Jass *et al.* in their WHO Blue Book classification [42] classified HPS as at least five hyperplastic polyps (HPs) proximal to the sigmoid colon, at least two of which are >1cm in size, or more than 30 HPs at any site in the large bowel. An alternate system from Rashid *et al. *[43] classified HPS as any person with greater than 20 hyperplastic polyps in their large bowel.

The precise molecular mechanism that underlies HPS has not currently been determined. Several studies have shown a variety of genetic changes within these hyperplastic polyps, including increased frequency of chromosomal 1p allelic loss [43], somatic *BRAF* [44] (80% of HP’s) and *k-RAS2* (10% of HP’s) mutations [44]. MSI has also been shown to be present at only low frequency in HP’s, at approximately 2% [44]. A recent study on HPS in the Ashkenazi Jewish population mapped a high penetrance gene, *CRAC1* to 15q13.3–q14 [45].

It has been shown that patients with the characteristics of HPS have an increased risk of developing colorectal carcinoma [46-48]. 

### The Somatic Genetic Pathways in Sporadic Colorectal Cancer

Using the classical colorectal cancer syndromes such as FAP and Lynch Syndrome as a paradigm, significant knowledge has been gained regarding the development of sporadic cases of colorectal cancer. Several pathways have been delineated, based on the molecular “signatures” of both colorectal cancer, and their precursor lesions.

### The Chromosomal Instability (CIN) Pathway

Fogel and Vogelstein [49] proposed a model of colorectal carcinogenesis suggesting that mutational activation of proto-oncogenes to oncogenes as well as mutational inactivation of tumour suppression genes in a stepwise sequence of events leading to colorectal carcinogenesis. 

In their model, it was suggested that mutation of the k-ras proto-oncogene leading to activation of the oncogene as well as mutational inactivation of tumour suppressor genes existing on chromosomes 5q (*APC*), 7p (*p53*) and 18q (*SMAD4*) were the key initiators of colorectal carcinogenesis. They also hypothesised that DNA hypomethylation, believed to have a role in the silencing and expression of genes, played a part in this mechanism. 

Several important genes have been characterised as being involved in the Chromosomal Instability pathway, namely the APC, k-ras, p53 genes and mutations in the TGF-β signalling pathway.

### Adenomatous Polyposis Coli (*APC*)

The *APC* tumour suppressor gene is thought to have a pivotal role in the initiation of CRC. It was first characterised in the germline of patients suffering from Familial Adenomatous Polyposis (FAP). The *APC* gene is located on chromosome 5q21 [50]. It encodes a 312 kDa protein composing of 2843 amino acids. The protein product has several functions and interacts with important proteins controlling cellular function including β-catenin, glycogen synthase kinase (GSK), end-binding protein (EB) 1 and Bub kinase [51-53]. The first two proteins are intimately involved in the *Wnt* signalling pathway.

*APC* is important in regulation of intracellular β catenin levels. β catenin binds to T-cell factor transcription factors and leads to activation of gene transcription. The wild type (non-mutated) *APC* protein product binds to β-catenin, GSK-3  β and CK1α/ β using an axin/conductin skeleton [54]. This leads to increased β-catenin breakdown by promoting phosphorylation of β-catenin leading to its degradation *via *the ubiquitin-proteasome pathway. The mutated form of APC cannot bind to β-catenin which accumulates [51, 55, 56]. 

When intracellular β-catenin levels rise as a result of faulty *APC* function, this promotes carcinogenesis through the *Wnt* pathway which is a key signal transduction pathway involved in the homeostasis of colonic epithelium. 

The *Wnt* family of glycoproteins are involved in activation of the *Wnt */  β-catenin pathway [57]. *Wnt* secretion is controlled by the Wntless/evenness transmembrane protein [58]. The Wnt glycoprotein binds to the transmembrane receptor frizzled and low-density lipoprotein receptor-related protein LRP-5/6 which leads to phosphorlation of LRP by GSK-3 β and CK1α thus causing recruitment of axin to the cell membrane [59]. 

Axin is then degraded preventing integration into the GSK-3β / β-catenin/axin complex and thus causing a rise in intracellular β-catenin levels [59]. This leads to stabilised β-catenin entering the nucleus of the cell, associating with TCF/LEF transcription factors and causing activation of downstream Wnt target genes. 

Mutant *APC* has been shown in rat models to allow nuclear accumulation of β-catenin at the base of normal colonic crypts leading to permanent stimulation of the *Wnt* pathway [60] which causes hyperproliferation of colonic epithelium, thus increasing the likelihood of dysplasia and progression to malignancy [61].

*APC* protein may play a role in the control of chromosomal division through the formation of microtubules [62] as well as a companion protein, EB-1, a protein associated with the C-terminal end of the *APC* protein [63]. Microtubules are important in cell division as they link to the kinetochore. Recent evidence shows that non-mutated APC protein helps to stabilise kinetochore microtubules and allows them to attach to the chromosomes [64].

The most common mutation in the *APC* gene is a premature stop codon caused by a point mutation leading to a truncuated protein [65]. Approximately 60% of point mutations occur within the mutation cluster region at the 3’ end of the *APC* gene [54]. *APC* mutations have been observed in 30-70% of sporadic adenomas and in 34-72% of sporadic carcinomas [65-68]. *APC* mutations have been shown to occur at a similar frequency at all stages of colorectal carcinogenesis, suggesting that they are an early event. 

However, allelic loss of *APC* has been shown to increase in frequency as there is progression towards carcinoma [69]. Adenomas as small as 0.5cm carry *APC* mutations [70], reinforcing the theory that these are initiators of a cascade of genetic changes leading to carcinogenesis.

### *K-ras* mutations

*K-ras* is an oncogene thought to play an important role in the colorectal adenoma-carcinoma sequcence. The oncogene codes for a 21kDa protein, ras p21, which is involved in signal transduction of regulatory pathways involved in cell differentiation [71, 72]. 

When expressed it is a GTP-binding protein located on the cytoplasmic aspect of the cell membrane and has intrinsic GTPase activity that is regulated by other proteins [73]. It has been found that all carcinogenic mutations of the ras oncogene affect codons in the domain responsible for GTP binding leading to a decrease in the GTPase activity, causing permanent activation of the ras protein [73]. This causes unconstrained cellular proliferation. 

Mutations in *k-ras* have been found in 35-42% of colorectal adenomas and carcinomas [74, 75]. These studies also demonstrated that *k-ras* mutation is more common as the size of the adenoma increased. 54 small benign lesions of the colorectum were analysed in a study by Jen *et al. *[75] who demonstrated that 82% of the adenomas in the group demonstrated *APC* mutations, but none of the hyperplastic polyps possessed this mutation. In both groups of polyps there was approximately a 22-25% rate of *k-ras* mutation but in dysplastic lesions this *k-ras* mutation was always associated with an APC mutation, suggesting that *k-ras* mutations may be insufficient on their own to initiate carcinogenesis. 

Biopsies of normal colonic mucosa in patients who have had resections for colonic carcinoma have shown a much increased frequency of *k-ras* mutations in the normal mucosa. It has been suggested by Zhu *et al. *[76] and Minamoto *et al. *[77] that this may be a potential marker for stratifying risk in patients with colorectal carcinoma. 

### p53

*p53* is known as the “Guardian of the Genome” because it blocks cell proliferation in the presence of DNA damage as well as promoting DNA repair and causing apoptosis if the repair is insufficient [78]. 

The *p53* gene is located on the short arm of chromosome 17 and was thought in initial cytogenetic studies to be responsible for the initiation of colorectal cancer [79, 80]. The relationship between *p53* and aneuploidy is more complex as studies have shown aneuploidy not associated with *p53* overexpression [81], chromosomal abnormalities in patients with normal (wild-type) *p53* [82], and mutant p53 in cells with normal chromosomal ploidy [83]. It is hypothesised that the reason for such large variations is that the different mutations have different effects on the tumour phenotype because of different effects on the downstream part of the *p53* pathway [84].

The mechanism of carcinogenesis with respect to *p53* and colorectal cancer is thought to be functional inactivation due to either mis-sense muations in the DNA binding domain of *p53* or oncogenic viral interaction with *p53* [85, 86].

These mutations lead to accumulation of *p53* in cells, as mutant *p53* is resistant to degradation by proteolysis (by the mdm-2-ubiquitin pathway) leading to accumulation in the cell [87]. Normal (wild-type) *p53* has a very short half life and thus does not persist. 

Functional inactivation or alteration of *p53* or allelic loss at 17p has been shown to be present in between 4-26% of colorectal adenomas [88, 89]. It is has also been shown in 50% of invasive foci in adenomatous polyps [89] and in 50-75% of adenocarcinomas of the colon [90-92]. It is hypothesised that functional inactivation of the *p53* protein is one of the factors necessary for the transition from adenoma – carcinoma [84] in the colon.

### 18q (*SMAD4*) Loss / TGF-β Pathway Mutations

Originally mutations in a tumour suppressor gene provisionally known as the “Deleted in Colorectal Carcinoma” (*DCC*) gene were thought to be one of the mutations responsible for initiation of colorectal carcinogenesis [93], because it was frequently lost in colorectal cancer and was found to be located on chromosome 18q.

Further analysis showed the *DCC* gene actually coded for a component of the receptor complex that mediated the effects of netrin-1, a molecule involved in axon guidance [94], which seemed an unlikely function for this role. In a study of 57 colorectal cancers, it was found that there were almost never any mutations in the *DCC* gene in human colorectal tumours showing 18q allelic loss [95]. 

Mouse knockout models of *APC*/*DCC* have shown that loss of the *DCC* gene in mice that have adenomas initiated by *APC* loss causes a bias towards highly dysplastic adenomas [96]. The *DCC* gene possesses pro-apoptotic activity, thought to be due to cleavage by a capsase (cysteine protease) exposing a pro-apoptotic domain on the *DCC* receptor, which is inhibited by netrin-1. Over-expression of netrin-1 in mouse models, coupled with loss of the *APC* gene has been shown to suppress pro-apoptotic activity and is postulated to promote carcinogenesis. 

However, studies of human colorectal cancers [96] have shown that only 7% of colorectal cancers have over-expression of netrin-1, implying that loss of the *DCC* receptor complex by 18q allelic loss or direct mutation may not confer the same selective advantage towards human colorectal cancer cells. 

Characterisation and identification of other tumour suppressor genes in the 18q region revealed two candidate genes, namely *SMAD2* and *SMAD4*. SMAD proteins are human homologs of the drosophila protein, mothers against decapentaplegic (MAD) and the C. elegans protein SMA.

*SMAD4* was originally identified as a candidate gene that is mutated in early pancreatic carcinoma [97]. The protein product of the *SMAD4* gene codes for intracellular mediators of the transforming growth factor (TGF) β pathway [98, 99]. This is an inhibitory pathway that is responsible for exerting a wide range of effects including regulation of cell growth, differentiation and apoptosis and has been implicated in a wide range of human cancers [99]. 

TGF-β signalling is initiated *via *the binding of TGF-  β to type II TGF- β (*TGFBR2*) receptors. The commonest isoform of TGF- β, TGFB1 binds to TGFBR2 which then leads to recruitment *via *phosphorylation of the type I TGF- β receptor (*TGFBR1*) leading to activation of TGFBR1 protein kinase. This causes phosphorylation of SMAD2 and SMAD3, two transcription factors that then bind to and activate SMAD4. These complexes then migrate to the nucleus where they activate a series of TGF- β responsive genes [100, 101]. These genes typically include the cell-cycle checkpoint genes *CDKN1A* (p21), *CDKN1B* (p27) and *CDKN2B* (p15), which when activated cause cell cycle arrest [102]. Therefore the TGF- β pathway acts as a tumour suppressor pathway in normal colonic epithelium.

In studies of colorectal carcinomas excised at surgery, mutations of the *SMAD4* and *SMAD2* genes have been observed [103-105]. A study by Zhou *et al. *[106] took human colorectal cancer cell lines and used targeted deletion to inactivate the SMAD4 gene. They found that this prevented transduction of the TGF- β pathway leading to uncontrolled cellular proliferation. The ubitquitin-proteasome pathway has been implicated as being responsible for accelerated breakdown of the mutated *SMAD2/4* protein product, again leading to uncontrolled cell proliferation [100].

Conversely, it has been found that at the later stages of colorectal cancer development, the TGF- β pathway actually acts to promote invasion and metastasis, shown in several experimental models where colonic epithelial cells were exposed to high levels of TGF- β which induced malignant transformation [107] as well as invasion causing metastasis [108]. This is thought to be due to the fact that TGF- β regulates the production of growth factors including TGF- β, FGF and EGF [101], as well as the fact that tumour cells at an advanced state of development become resistant to the inhibitory effect of the TGF- β pathway.

### The Microsatellite Instability Pathway (MSI)

Microsatellites, also known as simple sequence repeats are polymorphic loci present in all cellular DNA. They consist of tandem repeats, usually of between 1-4 base pairs in length, repeated many times [11]. 

Microsatellites are highly variable and prone to mutation, due to slipped strand mispairing (slippage) during DNA replication. Mutations in microsatellites are usually repaired by mismatch repair (MMR) enzymes, but when these repair enzymes have been inactivated by mutation of the gene encoding the MMR enzyme, the microsatellite mutations accumulate as DNA is replicated [11], leading to “microsatellite instability” (MSI). 

In cells that have inactivated MMR enzymes both microsatellite DNA and nucleotide repeat sequences in other key genes, such as cell cycle regulatory genes are at risk. It is on this basis that MSI is used a surrogate marker for the state of hypermutability or a “mutator phenotype” [109, 110].

In sporadic colorectal tumours, germline mutations of MMR genes are rare in tumours that are MSI-high, but lack of expression of *MLH1* has been found to be very common (up to 95%) in sporadic MSI+ tumours and is thought to be due to hypermethylation of the promoter region of *MLH1* gene [111].

MSI +ve tumours can be further sub-divided into MSI-high (where there is a high level of instability) or MSI-low (where there is a low level of instability). In a paper by Dietmaier [14] *et al.* MSI-high was defined as more than 20% of loci being unstable and MSI-low defined as less than 10% of loci being unstable in comparison between the germline DNA and the tumour itself. 

A recent study by Samowitz *et al. *[112] has only demonstrated MSI-high in 1.8% of all sporadic adenomas, rising to 2.5% in proximal adenomas. This is in contrast to patients with Lynch Syndrome, where almost all adenomas are found to be MSI-H.

One of the mechanisms hypothesised to cause progression from adenoma to carcinoma is mutation of mononucleotide repeats in the coding region for the TGF- β type II receptor (*TGFBR2*). This has been found to be mutated by inactivation due to hypermethylation of the promoter region in over 90% of colorectal cancers showing MSI [113]. In sporadic adenomas the earliest stage at which RII mutations could be detected was in high grade dysplastic adenomas. In adenomas with foci of invasive adenocarcinoma, it was noted that the *TGFBR2* had mutated in 75% of cases [114]. It has been suggested that mutation of TGF-β RII is a critical rate limiting step in the transformation from adenoma to carcinoma and that this mutation promotes this [84].

There have also been recent studies that have suggested “crosstalk” between the Wnt signalling pathway and the TGF-β signalling pathway [115, 116]. This is based on knockout mouse models that possess not only *APC* mutations but *SMAD*/TGF-β pathway progressing to larger, more dysplastic polyps at a much faster rate, suggesting that these activation pathways may be synergistic.

### The “Serrated Adenoma Pathway” 

Serrated adenomas are have variant of hyperplastic polyps. They usually occur in the right colon of middle aged females and have an increased risk of malignant transformation [117]. These polyps display alteration of their proliferative zones, with dilation of the crypts, occasionally extending into the muscularis mucosae. The crypts can also herniate through the muscularis mucosae, producing an appearance similar to invasive carcinoma [118]. Serration is also seen at the base of the crypts.

### *BRAF* Mutations

RAS proteins are involved in the *RAS-RAF-MEK-ERK-MAP* kinase pathway, involved in transducting cellular response to growth signals. As shown above, somatic mutations of the *RAS* gene (*k-ras*) can cause malignant transformation. There are three *RAF* genes that encode serine/threonine kinases that are regulated by binding to *RAS* [119, 120].

Mutations of the *BRAF* gene have been shown to be an alternative route for colorectal carcinogenesis [121]. In a study by Davies *et al. *[121] a panel of primary colorectal tumours and colorectal cancer cell lines were screened for mutations in *BRAF*. Over 10% of tumours & cell lines were found to have *BRAF* mutations. 

Mutations in *BRAF* [121] affect two regions of the *BRAF* kinase domain, namely the activation segment (which protects the substrate binding site) and the G loop (which mediates the binding of ATP). These mutated forms of *BRAF* have elevated kinase activity and are probably responsible for unregulated growth signalling and therefore the initiation of carcinogenesis. 

In a study by Chan *et al. *[122] “serrated” polyps were analysed for mutations in *BRAF* or *k-ras.* They found of these serrated polyps, *BRAF* mutations were found in 36% of hyperplastic polyps(HP), 20% of admixed hyperplastic polyp/adenomas(HP/AD) and 100% of serrated adenomas (SA). *k-ras* mutations were found in 18% of HP’s, 60% of HP/AD’s and 0% of SA’s. They also showed that 90% of serrated polyps that showed dysplasia had mutations in *BRAF* or *k-ras* and that these acquired mutations were mutually exclusive, i.e. either *BRAF* or *k-ras* was present.

*BRAF* mutation V600E is associated with somatic mismatch repair deficiency (MSI) and found in 40% of the cases while in mismatch repair proficient tumors (MSS) the frequency is around 5%. In sporadic MSI cases of colon cancer this mutation is found in proximal colon tumors with MLH1 methylation (80% of cases), while in tumors from hereditary nonpolyposis colorectal cancer (HNPCC) cases with MLH1, MSH2 or MSH6 germline mutations, no *BRAF* mutations are detected. Because of this it has been proposed that mutation of *BRAF* at V600E can be used as an exclusion criterion for Lynch Syndrome. 

### CpG Island Methylation

CpG island methylation is another phenomenon which may influence progression to colorectal carcinogenesis. DNA methylation is present at a low level in almost all colonic carcinomas, and it has been shown in a subset (known as the CPG Island Methylator Phenotype “CIMP”, discussed below) to occur at a much higher frequency [123].

CpG islands are regions of DNA where there are a large number of contiguous cytosine (C) and guanine (G) base pairs linked by a phosphodiester bond (hence the name “CpG”). These areas are found to exist in the promoter regions of genes involved in many functions, but in colorectal cancer the DNA repair enzyme *MLH1* is such a region [124]. Methylation of these promoter regions of these genes leads to silencing and therefore non-expression of the gene product, leading to the initiation of carcinogenesis. Other genes which such regions are involved in all types of sporadic cancer include *p16*, *MLH1* and *BRCA1* [125]. 

In normal colonic mucosa [126], methylation has been noted to increase in a linear manner proportional with age. It has also been noted in patients with MSI positive colorectal cancer [127] and caused by several carcinogens, with varying levels of methylation such as the tobacco-derived carcinogen 4-(methylnitrosamino)-1-(3-pyridyl)-1- butanone (16.7%), plutonium (81.8%) and X-rays (38.1%) [126].

Toyota *et al. *[124] carried out a study of the frequency of CpG island methylation in colorectal cancer lines. They found that there were two main distinct patterns of methylation in colorectal cancer, which they termed Type A (ageing specific) and Type C (cancer specific). 

In type A methylation, they found increasing global methylation in colorectal cancer lines in relation to age. In type C methylation, they noted what they termed a “hypermethylator phenotype”, termed CpG Island Methylator Phenotype positive (CIMP+). 

They found in cell lines that were CIMP+ there was widespread silencing of genes through promoter methylation which they hypothesised could also cause inactivation of the hMLH1 promoter and thus may play a role in up to 75% of sporadic colorectal carcinoma cases that are MSI+. They suggested that the mechanism underlying this was a loss of protection against methylation through an epigenetic error. 

Weisenberger *et al. *[123] further contributed to our knowledge of the CIMP+ phenotype by carrying out Methylight quantitive methylation specific PCR assays on a library set of colorectal carcinomas. They found that they could reliably identify tumours with the CIMP+ phenotype using a five gene set consisting of *CACNA1G*, *IGF2*, *NEUROG1*, *RUNX3* and *SOCS1*, known collectively as the “CIMP Panel”

A further study by Ogino *et al. *[128] studied colorectal carcinomas collected *via *the Nurses Health Study. They examined the methylation status of the CIMP panel, as well as two additional genes (*CDKN2A* and *CRABP1*) and the promoter region of *MGMT* (O-6-methylguanine-DNA methyltransferase), a gene involved in DNA repair. 

In tumours where 4/5 of the CIMP panel were hypermethylated, they found a decrease in the expression of nuclear p27 and p53, as well as reduced expression of *COX2* and increased *TGFBR2* mutations. They also found using their total panel of eight genes to look at promoter methylation that tumours with between 1-5 methylated genes and MSI-low have high levels of methylation of *MGMT*. They have termed this “CIMP-low” (as opposed to CIMP-high which is equivalent to CIMP+).

These findings suggest distinct molecular pathways for CIMP-high tumours, and possibly for CIMP-low tumours. The underlying cause of this type of mutation is still not clear, however the concept of “epimutagens”, substances that promote aberrant methylation [129] of promoter regions has been postulated as a possible mechanism. Grady [130] suggested that the epimutagen hypothesis would fit well with the concept of CIMP-low and CIMP-high, however the mechanisms and place of promoter methylation in colorectal carcinogenesis is still not fully understood.

## CONCLUSIONS

In summary, molecular advances over the last 30 years have led to an exponential increase in the understanding of the molecular mechanisms underlying colorectal carcinogenesis. However, there is a great deal of further work that needs to be done to clarify the precise mechanisms underlying colorectal cancer and its initiation and progression, most especially at the adenoma level, before progression to a malignant lesion occurs.
